# Ammonia-oxidizing archaea have similar power requirements in diverse marine oxic sediments

**DOI:** 10.1038/s41396-021-01041-6

**Published:** 2021-06-22

**Authors:** Rui Zhao, José M. Mogollón, Desiree L. Roerdink, Ingunn H. Thorseth, Ingeborg Økland, Steffen L. Jørgensen

**Affiliations:** 1 School of Marine Science and Policy, University of Delaware, Lewes, DE USA; 2 Institute of Environmental Sciences (CML), Leiden University, Leiden, the Netherlands; 3 Centre for Deep Sea Research, Department of Earth Science, University of Bergen, Bergen, Norway

**Keywords:** Microbial ecology, Biogeochemistry, Water microbiology

## Abstract

Energy/power availability is regarded as one of the ultimate controlling factors of microbial abundance in the deep biosphere, where fewer cells are found in habitats of lower energy availability. A critical assumption driving the proportional relationship between total cell abundance and power availability is that the cell-specific power requirement keeps constant or varies over smaller ranges than other variables, which has yet to be validated. Here we present a quantitative framework to determine the cell-specific power requirement of the omnipresent ammonia-oxidizing archaea (AOA) in eight sediment cores with 3–4 orders of magnitude variations of organic matter flux and oxygen penetration depth. Our results show that despite the six orders of magnitude variations in the rates and power supply of nitrification and AOA abundances across these eight cores, the cell-specific power requirement of AOA from different cores and depths overlaps within the narrow range of 10^−19^–10^−17^ W cell^−1^, where the lower end may represent the basal power requirement of microorganisms persisting in subseafloor sediments. In individual cores, AOA also exhibit similar cell-specific power requirements, regardless of the AOA population size or sediment depth/age. Such quantitative insights establish a relationship between the power supply and the total abundance of AOA, and therefore lay a foundation for a first-order estimate of the standing stock of AOA in global marine oxic sediments.

## Introduction

The deep sedimentary biosphere underneath the seafloor is estimated to host a similar number of microbes as found in the oceans [1, 2]. The spatial distribution of these microbial cells varies on a global scale: higher cell abundances are observed in sediments on continental margins with high organic matter contents [3], while lower cell abundances are detected in oligotrophic sediments underneath ocean gyres [1, 4, 5]. Microbial cell numbers tend to decrease vertically with sediment depth/age following a power-law [1, 2, 6]. The underlying reasons of this phenomenon have been attributed to several environmental factors, e.g., temperature, nutrients, distance from land, sedimentation rate, and organic matter flux [1, 2, 6], viral infection [7–9], and energy/power availability [10, 11]. Among these factors, the energy availability, mainly derived from organic matter degradation, has been argued as the most important factor governing the biomass in marine sediments [10, 12–14]. Most microbial cells in subseafloor sediments are subsisting under continuous energy limitation [14, 15] and are thought to be operating at their basal power requirement (BPR), defined as “the energy flux associated with the minimal complement of functions required to sustain a metabolically active state” [12]. From the energetic perspective, cell numbers of a certain functional group are controlled not only by the total power availability for this group but also the power requirement per cell (i.e., cell-specific power requirement). In order to have cell numbers proportional to the total energy availability over different spatial or temporal scales, one has to assume that the cell-specific power requirements (probably the BPR) of this group are similar over those scales. However, this critical assumption has not been sufficiently validated.

Quantification of the BPR of functional groups in natural environments is challenging, owing to the scarcity of concurrent, quantitative data describing the abundances and power availability of functional groups, the latter of which requires estimates of both Gibbs energy and reaction rate. There are only a handful of attempts of estimating BPR focused on the bulk community rather than individual functional groups, by assuming that all cells are involved in aerobic H_2_ oxidation (knallgas reaction) [10] or particulate organic matter degradation [11, 14, 16]. However, most microbial dwellers in marine sediments are uncultured and only distantly related to cultured representatives [17], and thus their metabolisms (e.g., substrates and products) are still largely unknown and likely to be more diverse than previously assumed. This is demonstrated even in oligotrophic sediments where microbial communities of small population size and low diversity were detected [18–23]. In these sediments, a significant proportion of microbial cells (e.g., the autotrophic ammonia-oxidizing Thaumarchaeota, see below) may not be directly involved in the above-mentioned processes. Therefore, a better understanding of the energy catabolism and absolute abundances of functional groups is essential to correctly constrain their energy requirement in the natural environment.

Ammonia-oxidizing Archaea (AOA, affiliated to the phylum of Thaumarchaeota) are omnipresent in marine oxic sediments [18–23], which covers a significant proportion of the global seafloor [5]. The metabolism of AOA exerts a profound influence on the biogeochemistry of marine sediments by linking the gain and loss of bioavailable nitrogen [24–26] and contributing significantly to benthic primary production [27–29] due to their chemoautotrophic lifestyle. AOA is also an exceptional functional guild well-suited for quantitative cell-specific energetic analysis, due to their (i) known core metabolic pathways for energy conservation (i.e., aerobic ammonia oxidation [30, 31]), (ii) distinct molecular markers (*amoA* gene encoding the ammonia monooxygenase alpha subunit) for abundance quantification, and (iii) clear geochemical imprints (oxygen consumption and nitrate accumulation when coupled with nitrite oxidizers) for reaction rate estimation.

Here we present quantitative insights into the bioenergetics of AOA in marine oxic sediments in eight different cores from five oceanographic regions, with sediment depths spanning from the top millimeters to 42 meters below seafloor (mbsf). We calculate nitrification rates using a reaction-transport model that simulates the measured porewater profiles of oxygen, ammonium, nitrate, organic, and inorganic carbon [13]. We also calculate the Gibbs free energy and power supply of nitrification based on the modeled profiles of relevant chemical species. In addition, we derive a power-law relationship between the percentage of AOA in the total community and the relative depth in the oxic zone (i.e., the depth divided by the total thickness of the oxic zone), and then use that relationship to estimate AOA abundances based on total cell numbers. The combination of these quantitative data makes it possible to calculate the cell-specific power requirement of AOA over a wide spectrum of sediment depths/ages, and to explore the BPR of AOA in the deep sedimentary biosphere.

## Materials and methods

### Study sites description

**Fig. 1:**
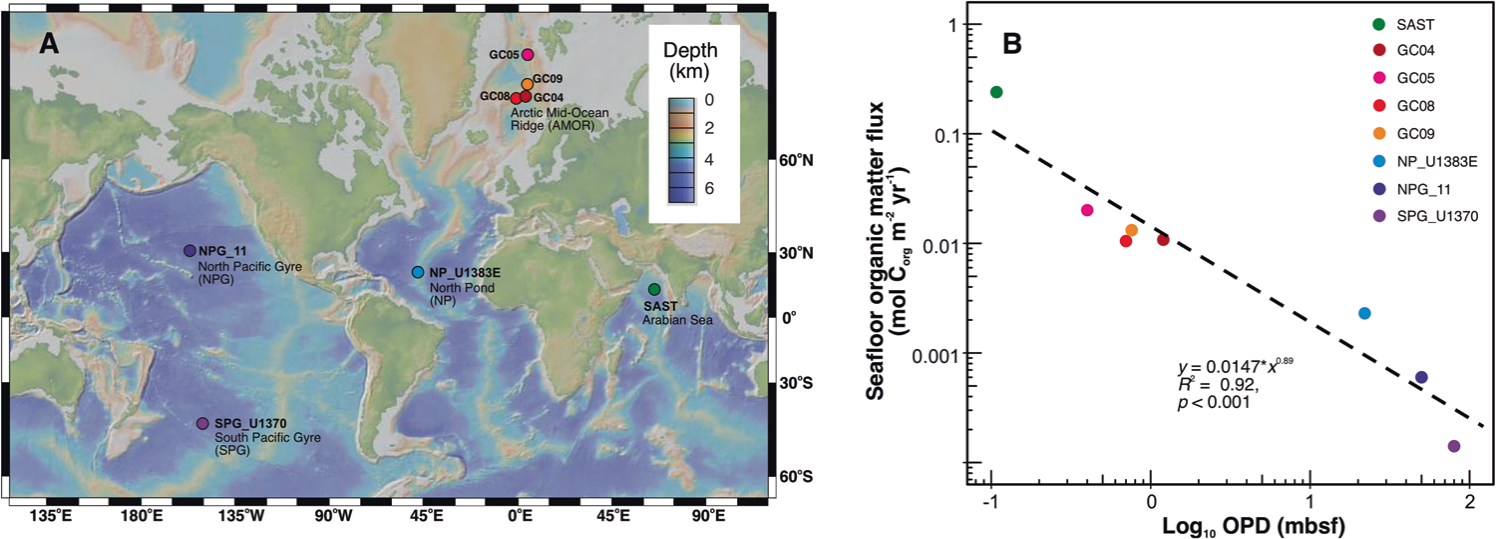
Locations and geochemical property variations of the sediment cores investigated in this study. (**A**) A global bathymetric map showing the locations of the sediment cores. Inset in upper right indicates seafloor depth in kilometers. (**B**) Variations of seafloor organic matter flux and oxygen penetration depth (OPD) in the sediment cores. Organic matter flux, the amount of organic matter deposited to the seafloor per area and time (in the unit of mol C_org_ m^−2^ yr^−1^), was constrained by the reaction-transport model. OPD was set at the depth where O_2_ concentration drops below 1 µM. The dashed line denotes the power-law correlation between OPD and seafloor organic matter flux. Other sediment properties of each site are included in Table 1.

We studied eight sediment cores from the following five oceanographic regions: one sediment core from the Southern Arabian Sea station (SAST) [32, 33], four gravity cores (GC04, GC05, GC08, and GC09) from the Arctic Mid-Ocean Ridge (AMOR) in the Norwegian-Greenland Sea [13], one piston core from North Pond beneath the North Atlantic Gyre (NP_U1383E, [23, 34, 35]), one core from the North Pacific Gyre (NPG_11; [4, 36]), and finally one core from the South Pacific Gyre (SPG_U1370; [5]) (Fig. 1A and Table 1). The locations of these sites are shown on a global bathymetric map prepared using GeoMapApp v3.2.1 using the default Global Multi-Resolution Topography Synthesis [37] basemap. We selected these cores because of the access to the following two types of data: (i) geochemical data, especially the depth profiles of oxygen, ammonium, and nitrate, and dissolved inorganic carbon (DIC) in the porewater, as well as the total organic carbon contents (TOC) in the solid phase (Table S1), which are essential to calibrate the reaction-transport model (see below), and (ii) available microbiological samples or total cell counts data that are a prerequisite to estimate AOA abundances (Table S1). All geochemical profiles and total cell abundance quantification data were published previously, except for the AOA *amoA* gene abundances in the four AMOR cores, which were collected in this study following the procedure described below. We also provide below detailed descriptions on the measurements of porewater nitrite and pH of the AMOR cores. For details about the sample collection, geochemical measurement, and microbiological analyses, readers are advised to consult the original publications (listed in Table S1).

**Table 1:** Properties of sediment sites considered in this study.

Site	Sediment core ID	Coring method	Latitude	Longitude	Water depth (m)	Organic matter content (%)	Sedimentation rate (m/yr)
Arabian Sea	SAST	Multiple-coring	10°02'	65°00'	4,424	0.05–0.4	1.2E−5
Arctic Mid-Ocean Ridge(AMOR)	GC04	Gravity coring	72°16'	1°42'	2,668	0.3–1.0	2.0E−5
	GC05	Gravity coring	76°55'	7°7'	3,007	0.3–1.8	2.5E−5
	GC08	Gravity coring	71°97'	0°10'	2,476	0.3–0.6	2.0E−5
	GC09	Gravity coring	73°70'	7°34'	1,653	0.2–0.5	5.0E−5
North Pond (NP)	NP_U1383E	Piston coring	22°48'	−46°03'	4,476	<0.3	9.4E−6
North Pacific Gyre (NPG)	NPG_11	Multiple-coring, gravity coring, and piston coring	30°21'	−15°75'	6,000	--	1.0E−6
South Pacific Gyre (SPG)	SPG_U1370	Piston coring	−41°51'	−153°06'	5,075	0–0.25	1.5E−06

-- Not available.

### Porewater analysis for the AMOR cores

Porewater samples were extracted from selected depths throughout the recovered AMOR cores using 0.2 µm Rhizon filters, and the concentrations of ammonium, nitrate, and nitrite were determined onboard by photometric methods using a 4-channel Quaatro Continuous Flow Analyzer (Seal Analytical Ltd, Southampton, UK), with a detection limit of 0.1 µM for all species. Ammonium was determined using the indophenol method [38]. Nitrite was measured as a pink complex after reacting with *N*-1-naphthylethylenediamine dihydrochloride and sulfanilamide (i.e., Griess Reagent method). The sum of porewater nitrate and nitrite was measured using the same method, after converting nitrate to nitrite by a Cu−Cd reduction coil [39]. The difference between these two measurements was taken as the nitrate concentration. The pH measurements for the AMOR sediment cores were performed onboard using a Metrohm 826 pH mobile pH meter in a closed system to avoid outgassing of CO_2_. Calibration of the system was done daily using Metrohm disposable buffers (pH 4, 7, and 9). The pH in core U1383E of North Pond was determined onboard by an ion-selective electrode and reported in [40].

### Reaction-transport model simulation

We used the one-dimensional reaction-transport model described in [13, 41] to simulate the depth profiles of relevant solutes in the porewater and organic carbon content in the solid phase and to calculate the catabolic rates of various reactions in the eight cores. The explicitly modeled species include oxygen, nitrate, ammonium, Mn(II), and DIC in the pore water, and the TOC and MnO_2_ in the solid phase of sediments. The model considers two sets of reactions: (i) the primary reactions involved in organic matter degradation: aerobic degradation (*R*_1_), heterotrophic denitrification (*R*_2_), and MnO_2_ reduction (*R*_3_); (ii) and the secondary reactions including nitrification (*R*_4_), Mn(II) oxidation with oxygen (*R*_5_) and anaerobic ammonium oxidation (*R*_6_). For simplicity, *R*_3_, *R*_5_, and *R*_6_ were not considered for the three cores in which the considered sediment domain is fully oxic (i.e., NP_U1383E, NPG_11, and SPG_U1370). The model simulations assume that the geochemical profiles, including all implicit reactive intermediates, are at a near steady state.

Organic matter in the model was regarded to consist of three discrete components (the so-called 3-G model; [42]), with the first two as the reactive ones while the third one as non-reactive. Aerobic respiration (*R*_1_) was considered as the most favorable pathway of organic matter consumption, followed by heterotrophic denitrification (*R*_2_), and MnO_2_ reduction (*R*_3_). The secondary reactions (*R*_*4*_*−R*_*6*_) were represented by bimolecular kinetics. For the *C*/*N* stoichiometry of the degraded organic matter in the model, we used the measured values (Fig. S1) for the four AMOR cores. We assumed the Redfield ratio for the other cores because at these sites the ratio of nitrate to oxygen concentration (NO_3_^−^/−O_2_) in sediment porewater, a useful proxy of *C*/*N* ratio of the degraded organic matter in marine sediments [43, 44], were calculated to be indistinguishable from the Redfield ratio (0.094, [45]) determined in the ocean (0.098 ± 0.005 in SPG_U1370 and NPG_11 [36] and ~0.091 in North Pond sediments [35]).

For boundary conditions (Table S2), the model is constrained by fixed concentrations of O_2_, NO_3_^−^, DIC, and fixed organic matter flux at the sediment−water interface, and the absence of a gradient condition at the lower boundary of the sediment domain. The rest of the model parameters (Table S3) were calibrated by comparing the model simulation outputs against the measured depth profiles of O_2_, NO_3_^−^, NH_4_^+^, DIC, and TOC, if available, until satisfactory visual fits for all profiles were reached.

To assess the goodness of our model simulations, we calculated the root mean square error (RMSE), the square root of the sum of the squared differences between modeled and measured values, for O_2_, Mn(II), NO_3_^−^, NH_4_^+^, and DIC, if available (Table S4).

### Gibbs free energy and power supply calculation

The Gibbs free energy was calculated for nitrification (NH_4_^+^ + 2O_2_ = NO_3_^−^ + H_2_O + 2H^+^), by following the procedure described in [10], using the equation

$$\Delta G_{\mathrm{r}} = \Delta G_{\mathrm{r}}^0 + RT\ln Q_{\mathrm{r}}$$

where ∆*G*_r_^*0*^ and *Q*_r_ refer to the standard molar Gibbs energy and the reaction quotient of the indicated reaction, respectively, *R* represents the gas constant, and *T* denotes temperature in Kelvin. In this study, *∆G*_r_^*0*^ was calculated using the thermodynamic data of standard Gibbs energy of formation of each species and corrected to near in situ pressure and temperature (explicitly modeled in the above-described reaction-transport model), using the *R* package CHNOSZ [46]. *Q*_r_ stands for the reaction quotient, which can be calculated as a product of the activities of the reactants and products using the equation

$$Q_{\mathrm{r}} = {\mathrm{{\Pi}}}(a_i^{v_i})$$

where a_*i*_ is the activity of species *i* and *ν*_*i*_ is its stoichiometric coefficient (positive values for products and negative values for reactants). a_*i*_ is the product of a chemical species concentration [*i*] and its activity coefficient *γ*_*i*_, which was computed as a function of temperature and ionic strength by using an extended version of the Debye−Hückel equation [47]. Because the model simulations match well with the measured profiles, we used the simulated profiles in the Gibbs energy calculation to obtain a continuous prediction of ∆*G*_r_ with sediment depth. Since NH_3_ is a more likely substrate of ammonia-oxidizing archaea [48], we used the concentration of NH_3_ rather than NH_4_^+^ in this calculation. We estimated NH_3_ concentrations from the porewater NH_4_^+^ concentrations based on the pH-sensitive NH_3_/NH_4_^+^ equilibrium [49] using the equation

$$[{\mathrm{NH}}_{3}] = [ {\mathrm{NH}}_{4}^{+}] \times 10^{({\mathrm{pH}}- {\mathrm{pKa}})}$$

where pKa was taken as 9.3 [50], and pH in the individual cores from AMOR was taken as the average of the measured values in the core (Fig. S2). For the other cores where pH data were not reported, the average value (7.79) of all the measured pH in the AMOR samples was used. The final values were expressed in the unit of kJ per mole of electron transferred, kJ (mol e^−^)^−1^.

Following ref. [10], the power supply of nitrification in the marine oxic sediments, *P*_s_, is calculated using the following equation

$$P_{\mathrm{s}} = \Delta G_{\mathrm{r}} \ast R$$

where ∆*G*_r_ is the Gibbs energy of nitrification, *R* is the nitrification rate (in the unit of mol N m^−3^ yr^−1^), taken as the modeled nitrification rates. The resulting *P*_s_, in the unit of kJ m^−3^ yr^−1^, was converted to the unit of Watt m^−3^, given that 1 Watt is equal to 1 Joule per second (1 J/s).

### AOA abundances quantification in the AMOR cores

Total DNA in sediment horizons of the four AMOR cores was extracted as described in [13]. AOA abundances were quantified by targeting the archaeal *amoA* gene following the procedure described in [23], with qPCR efficiencies in the range of 92–97%. In addition, archaeal and bacterial 16S rRNA genes were quantified as described in [23]. Total cell abundances in the AMOR cores and NP_U1383E were estimated from the sum of archaeal and bacterial 16S rRNA gene copies reported previously [13, 23], assuming only one copy of 16S rRNA gene is present in each prokaryotic genome. This calculation is different from those used previously [13, 23], but matches with our genome data from the AMOR and North Pond sediments in which only a single copy RNA operon was observed in the recovered microbial genomes (e.g., [13, 31]).

### AOA abundance estimation for the other cores

For sediments from the NPG, SPG, and Arabian Sea where AOA abundance data are not available, we estimated the AOA abundances from the total cell counts, based on the power-law distribution of the relative abundance of AOA with sediment depth derived from the compilation of the amplicon sequencing datasets from AMOR (GC04, GC05, GC08, and GC09 [13]), and NP_U1383E [23] and two unpublished cores from AMOR. For each sediment sample, 16S rRNA gene amplicon was generated using the primer pair 515F/806R, and sequenced on an Ion Torrent Personal Genome Machine, as described in [13, 23]. The sequencing data were processed, including quality filtration, trimming, OTU clustering, and classification, following the procedure described in [23]. The relative abundance of AOA in each sediment horizon was calculated as the sum of the OTUs assigned to the family of Nitrosopumilaceae [23, 31]. To describe the vertical distribution pattern of AOA in the oxic zones, we normalized this zone in each core irrespective of the real depth (i.e., calculated the relative depth as the sediment depth divided by the oxygen penetration depth (OPD)) so that 0 corresponds to the surface and 1 to the boundary between oxic and anoxic sediments. The regression relationship between the fraction of AOA in the total microbial community and the relative depth was fitted with an exponential equation in MatLab using the “Curve Fitting” function (MATLAB and Statistics Toolbox Release 2019b, The MathWorks, Inc.). Based on this regression, the abundances of AOA in each core were calculated from the total cell numbers (either the total cell counts or qPCR-derived abundances; see Table S1 for the data sources).

### Cell-specific power requirement calculation

Cell-specific power requirement of AOA was calculated by dividing the power supply of nitrification by the AOA abundance estimated from the total cell numbers, assuming all the AOA cells are equally active (i.e., they have similar power requirements) at a particular site and contribute equally to the nitrification activity. To assess how low the power requirements of the sedimentary AOA are, we compared their cell-specific power requirements with values of various AOA strains cultured in the laboratory, including *Nitrosopumilus maritimus* SCM1 and NAOA6 incubated under various phosphate-limited conditions, [29], *N. adriaticus* NF5 and *N. piranensis* D3C reported in [51], as well as *N. cobalaminigenes* HCA1, *N. oxyclinae* HCE1, and *N. ureiphilus* PS01 reported in [52]. We calculated the cell-specific power requirements of these AOA strains as the product of the cell-specific nitrification rates (0.1–1 fmol N cell^−1^ h^−1^ [29, 51]) and the Gibbs energy of nitrification. Because the concentrations of the relevant reactants of nitrification were not reported in the above-mentioned studies, we used the range of the Gibbs energy of nitrification obtained in this study (e.g., −50 to −80 kJ (mol e^−^)^−1^) for the calculation. For comparison, we also included the cell-specific power requirement of AOB *Nitrosomonas marina*, 2.8 × 10^−14^ W cell^−1^, calculated by [10, 53] based on the chemostat experiment performed by [54].

## Results and discussion

### Geochemical profiles and reaction rates modeling

**Fig. 2:**
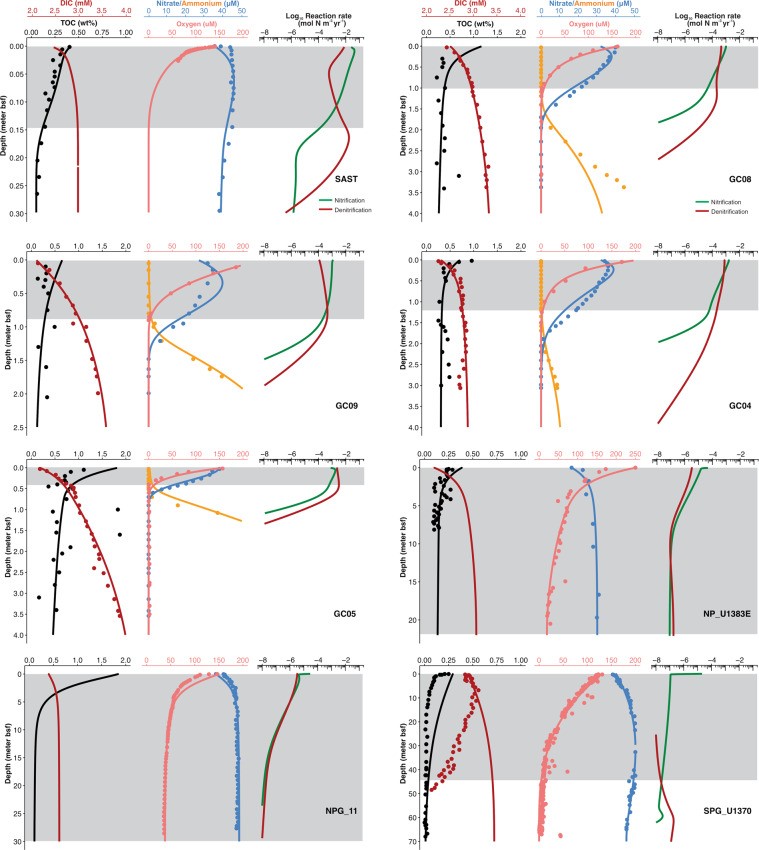
Depth profiles of chemical constituents and reaction rates of nitrification and denitrification in marine oxic sediments. Dots are measured values (the data sources were listed in Table S1), while lines represent simulation results from the reaction-transport modeling using the boundary conditions and model parameters listed in Tables S2 and S3, respectively. The shadowed area in each core indicates the oxic zone where Gibbs energy and power supply of nitrification were calculated. Note that different vertical scales are used for different cores. The steep drops of nitrification rate at the sediment surface in cores from NP, NPG, and SPG may result from the 0.01 µM (10 nM; Table S4) of ammonium concentration used for the boundary condition, which may overestimate the in situ ammonium concentrations in the sediments of these sites. TOC total organic carbon; DIC dissolved inorganic carbon.

All sediment cores considered in this study (Fig. 1A and Table 1) were retrieved from the seafloor at water depths greater than 1,600 meters in the open ocean, and can be classified as oligotrophic sediments based on the measured total organic carbon contents (TOC) of <0.7% (Fig. 2). Other important properties of the study sites are provided in Table 1. Despite being in different vertical scales, all cores exhibit the same characteristics (as shown in Fig. 2) and can be summarized as follows: (i) oxygen concentration monotonically decreases with depth due to the oxygen consumption coupled to sedimentary organic matter mineralization and the oxidation of reduced substances (e.g., Mn^2+^) from deeper anoxic sediments (especially in the four AMOR cores); (ii) nitrate concentration in the pore water increases beneath the sediment−water interface. Similar nitrate profiles were extensively observed in abyssal sediments (>2000 m water depth) [5, 20, 21, 33, 35, 41, 43, 55–60] and have been interpreted as the result of benthic nitrification, in which nitrate is produced from the oxidation of ammonium derived from organic matter mineralization.

The modeled concentrations of O_2_, NO_3_^−^, DIC, and TOC generally match well with the measured ones (Fig. 2) (see also model parameters and boundary conditions in Supplementary Tables S2, S3, and uncertainty shown as RMSE in Supplementary Table S4), and thereby provide quantitative constraints on the rates of various redox reactions occurring in these oxic sediments (e.g., nitrification and denitrification rates in Fig. 2). Our model can well reflect the turnover of ammonium in the oxic sediments (Fig. 2), which did not accumulate in the porewater. This indicates that its consumption rate (i.e., nitrification) is almost equal to its production rate (i.e., particulate organic nitrogen degradation) which is well constrained by the profiles of O_2_ and DIC (Fig. 2). It is worth noting that, owing to the gravity and piston coring methods used for some cores that are inefficient to recover the most surface sediments without disturbances (see Table 1 for the coring methods), the modeling results probably only reflect the biogeochemical processes in the recovered portion of the sediment cores.

Nitrate concentration is largely controlled by the balance between nitrification and denitrification. In some cores (e.g., the four AMOR cores and SAST), we adopted values of 4–30 µM for k_O2_ (Table S3), the oxygen inhibition constant on denitrification, to allow denitrification to occur in the oxic zone to account for the decreasing trend of the measured nitrate profiles in that zone. These values are consistent with those used in previous sediment diagenetic models (e.g., [61, 62]). Taking core GC08 as an example, we also performed a sensitivity analysis of k_O2_, by decreasing the oxygen inhibition constant by 10- and 100-fold. Our results suggested that using lower k_O2_ (i.e., 0.5 or 0.05 µM) would eliminate denitrification in the oxic zone, but make the simulated nitrate profile deviate substantially from the measured one (Fig. S3). Denitrification has been inferred [63, 64] and detected [65, 66] in bulk oxic marine sediments, which is mostly likely occurring in anoxic microniches in the bulk oxic environment, presumably similar to those in seawater [67]. However, there are still some poor matches observed between the modeled and measured concentration in the uppermost sediments in some cores (Fig. 2), which may indicate that the denitrification process in these sediments was not well represented by the model.

Organic matter flux across the sediment−water interface predicted by the model varies over four orders of magnitude in these cores (Fig. 1B and Table S2), with the highest (0.24 mol C_org_ m^−2^ yr^−1^) in SAST and the lowest (1.4 × 10^−4^ mol C_org_ m^−2^ yr^−1^) in SPG_U1370 (Table 3). Likewise, OPD, defined as the depth where O_2_ concentration drops below 1 µM) varies over three orders of magnitude at different locations (Figs. 1B and 2), with the shallowest predicted in SAST (0.15 mbsf) and deepest in SPG_U1370 (penetrating the entire sediment column, i.e., OPD > 67 mbsf). The organic matter flux exhibited a log−log decreasing relationship with the OPD (Fig. 1B), in accordance with observations from the Clarion−Clipperton Fracture zone [60]. The three to four orders of magnitude variations of organic matter flux and OPD across these sediment cores suggested that they could represent a wide range of marine oxic sediment settings.

Modeled nitrification rates generally decrease with sediment depth in individual cores (Fig. 2). Nitrification rates also exhibited seven orders of magnitude differences among the cores, with higher rates predicted in those cores with higher organic flux and shallower OPD (Fig. S4), probably because the direct substrates of nitrifiers, oxygen, and ammonium, are largely controlled by the rate of organic matter degradation. In particular, the highest nitrification rates in the oxic zone were predicted in SAST, with rates in the range of 10^−3^–10^−1^ mol N m^−3^ yr^−1^ (Fig. 2). The nitrification rates predicted in the AMOR cores vary between 10^−4^ and 10^−3^ mol N m^−3^ yr^−1^. The lowest rates were predicted in the SPG_U1370, with rates in the deep sediment in the range of 10^−8^–10^−7^ mol N m^−3^ yr^−1^ (Fig. S4). Nitrification rates in pelagic sediments have not been directly measured yet, but the modeled nitrification rates in our cores are at least five orders of magnitude lower than those measured in coastal sediments (4.7−58 × 10^4^ mol N m^−3^ yr^−1^ in North Sea coastal sediments; [68, 69]). Comparing with nitrification rates in the deep ocean, the modeled nitrification rates in all cores except for SAST are also at least two orders of magnitude lower than those measured in bathypelagic seawater in the North Atlantic Gyre (on the order of 10^−1^ nmol N L^−1^ d^−1^, [70]). These low nitrification rates underscore the relevance of numerical modeling in constraining sluggish reaction rates in low-energy settings such as subseafloor sediments.

### Gibbs free energy and power supply of nitrification

**Fig. 3:**
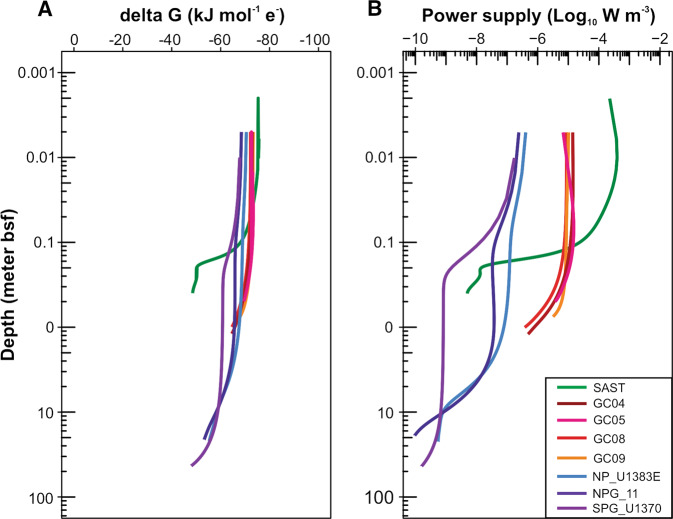
Gibbs free energy and power supply of nitrification in the investigated sediment cores. Both Gibbs energy (**A**) and power supply (**B**) of nitrification are calculated using the modeled concentrations of relevant species from the reaction-transport model simulation, as presented in Fig. 2. Note that sediment depths on the *y*-axis were log-transformed. See Supplementary Fig. S7 for the plots with linear sediment depth scales.

Given that nitrite, the intermediate compound of nitrification, was not observed to accumulate in any of the oxic sediments but only detected in some anoxic layers (<1 µM in GC04, (Fig. S5)), we assume that complete nitrification is dominating in the oxic zone of these sediments, i.e., ammonium is completely oxidized to nitrate. Gibbs energy for complete nitrification, ∆*G*_r_, in the unit of kJ (mol e^−^)^−1^, was calculated for all cores based on the modeled pore-water profiles. Although in some cores (e.g., GC09 and GC05) the modeled NH_4_^+^ concentrations in the oxic sediments are up to one order of magnitude higher than the detection limit (Fig. S6), such overestimates should only have negligible influence on the Gibbs free energy calculation, because the latter is linear with the logarithm of the former ([71] and Fig. S6E). Our Gibbs energy calculation shows that in every core nitrification is thermodynamically favorable throughout the oxic zone, with ∆*G*_r_ varying in the range of −80 to −50 kJ (mol e^−^)^−1^ (Fig. 3A; see also Fig. S7 for the Gibbs energy of nitrification plotted against the linear sediment depth scale). Although the Gibbs energy generally decreases with sediment depth in individual cores, it still varies within the same order of magnitude and therefore shows variations less pronounced than those of nitrification rates and organic matter flux (Fig. 3A). The calculated values of the energy availabilities of nitrification in these oxic sediments are much higher than the “biological energy quantum” (i.e., the lowest amount of energy that can be conserved by an organism, approximately −10 kJ (mol e^−^)^−1^; [72]), suggesting that nitrifiers could be well supported by the endogenous nitrification reaction.

We calculate the power supply of nitrification as the product of nitrification rate and ∆*G*_r_. Power supply of nitrification generally decreases with depth in individual cores (Fig. 3B), and exhibits a similar vertical variation pattern as the nitrification rate rather than the Gibbs energy of nitrification. This observation suggests that most variation of power supply can be attributed to the reaction rate. The power supply of nitrification in core SPG_U1370 is similar to that of microbial degradation of POC (~10^−15^–10^−12^ W cm^−3^; [11]). In the whole dataset, nitrification power supply varied substantially (in the range of 10^−10^–10^−4^ W m^−3^, Fig. 3B) in sediment cores with contrasting sediment properties reflected by the seafloor organic matter fluxes (Fig. 1B).

### AOA abundances in oxic sediments

**Fig. 4:**
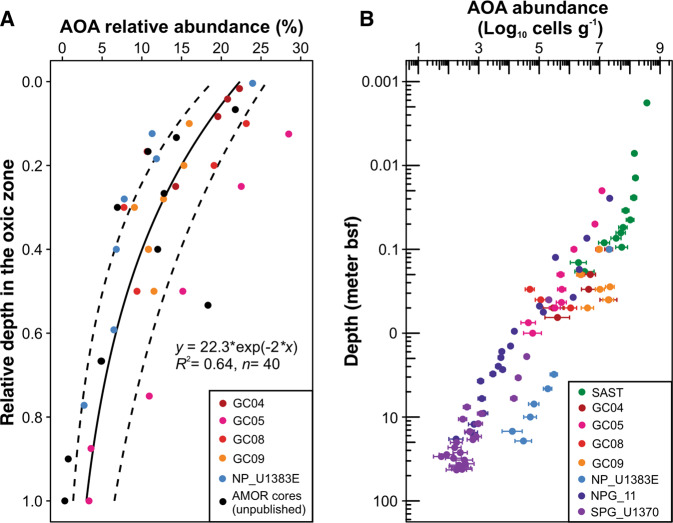
Abundances of ammonia-oxidizing archaea (AOA) in marine oxic sediments. (**A**) AOA relative abundance in the total microbial community as a function of their relative depth in the oxic zone of individual cores. The AOA relative abundances in the total microbial community were assessed by 16S rRNA gene amplicon sequencing. The solid line represents the best-fit power-law line, while the dashed lines denote the 95% confidence interval. (**B**) AOA absolute abundance as a function of sediment depth. The abundances were calculated as the product of the total cell numbers (derived from either cell counts or qPCR of 16S rRNA genes) and the relative abundance of AOA in the total communities present in (**A**). The error bars represent 95% of the confidence interval, derived from the relative abundance estimation shown in (**A**).

AOA are the most abundant archaea in marine oxic sediments, as shown in some of the investigated sediment sites (e.g., North Pond [23], AMOR [13, 73], and SPG [18, 19]) and also other locations [20–22]. Given that complete ammonia oxidizers seem to be absent [74] and ammonia-oxidizing bacteria are rarely detected in marine oligotrophic sediments [23], AOA are likely the predominant ammonia oxidizers in this environment. AOA in oligotrophic marine sediments are mainly dominated by the Eta and Upsilon clades of Nitrosopumilaceae [23, 31, 75] based on the 16S rRNA phylogeny [71]. In the absence of cultured representatives of these clades, obtaining their absolute abundance in marine sediments is a prerequisite to get more in situ physiological insights. Several discrete studies have evaluated the percentage of AOA in the total communities in marine sediments [20, 23, 73] and resulted in highly divergent estimates (<3–80% in different depths). However, systematic evaluation of the distribution of AOA in marine oxic sediments is still lacking. Here we compiled the datasets of AOA relative abundance in the four AMOR cores [13], one core from North Pond (NP_U1383E; [23]), and two other cores from AMOR (unpublished data), all of which were generated using the same experimental protocol (i.e., the same DNA extraction procedure, sequencing technology, and sequencing data processing strategy) to minimize potential methodological biases. Considering that the sediment cores considered in this study vary substantially in multiple properties and represent different sediment depths and age spans, we converted the sediment depths to the relative depths in the oxic zone (ranging from 0 to 1) to consider them in the same vertical domain. We observed a power-law relationship between the relative abundance of AOA and their relative depth in the oxic zone (*y* = 22.3*exp(−2*x*), *R*^2^ = 0.64, *n* = 40; Fig. 4A). This trend is observed in multiple cores with OPD ranging from millimeters to 22 mbsf, suggesting this might be a widespread phenomenon in marine oxic sediments. It is worth noting that the depth variation of AOA relative abundance in marine oxic sediments might not be solely controlled by oxygen concentration, because (i) oxygen of >1 µM was not known to directly affect AOA (e.g., [76]), and (ii) AOA percentage in the total communities is also affected by the population turnover of the surrounding communities that could have various but largely unknown responses to oxygen concentration changes. We acknowledge that this relationship is based on data from a small number of cores; future surveys of more sediment cores from geographically different locations may improve this estimation. It is also important to note that this relationship could not be extrapolated to the basal part of the oxic zone (i.e., the oxic−anoxic transition zone) because AOA showed elevated abundances probably resulting from in situ growth in this geochemical transition zone [23].

To examine whether the above-described regression can accurately predict the absolute abundance of AOA, we applied this regression to calculate the absolute abundances of AOA in the four AMOR cores and core NP_U1383E, and compared them with the abundances obtained from the qPCR of AOA *amoA* gene in these cores (Fig. S8). The absolute abundances of AOA obtained from the two methods matched well in NP_U1383E and showed <10-fold of differences in the four AMOR cores (Fig. S8). The relatively high accuracy for our dataset suggests that this empirical relationship provides a means to estimate the absolute abundance of AOA in marine oxic sediments without arbitrary assumptions.

We applied this regression to all the eight cores to estimate the AOA abundances from the total cell numbers. Similar to the reaction rate and power supply of nitrification, AOA absolute abundance in these cores varies over six orders of magnitude with higher abundances in the cores with higher organic flux and shallower OPD (Fig. 4B): the highest AOA abundances (1.5 × 10^7^−1.7 × 10^8^ cells cm^−3^) were estimated in SAST, intermediate in the AMOR cores, and the lowest in the SPG_U1370 (1.0 × 10^2^−1.0 × 10^5^ cells cm^−3^) (Fig. 4B). AOA abundances in sediments deeper than 1 mbsf are generally within the range of their abundance in bathypelagic seawater (10^1_^10^4^ cells cm^−3^, [77]), but are much higher in shallower sediments (10^5^−10^8^ cells cm^−3^, Fig. 4B).

The calculated AOA abundance also shows a log−log decreasing relationship with sediment depth across the eight cores (Fig. 4B), resembling that of the total cell numbers in the global marine sediments [1]. This calculation suggests that the size of the AOA population in marine oxic sediments, like the bulk microbial community, is controlled by the available power ultimately derived from organic matter degradation. AOA are also known to be capable of using alternative substrates such as urea and cyanate [31, 78, 79]. Regardless of which substrate(s) the sedimentary AOA use, most substrates of AOA are ultimately derived from the degradation of organic matter, which could explain the depth-dependent distribution of AOA in oxic sediments.

### Similar cell-specific power requirement of AOA from different sediment sites

**Fig. 5:**
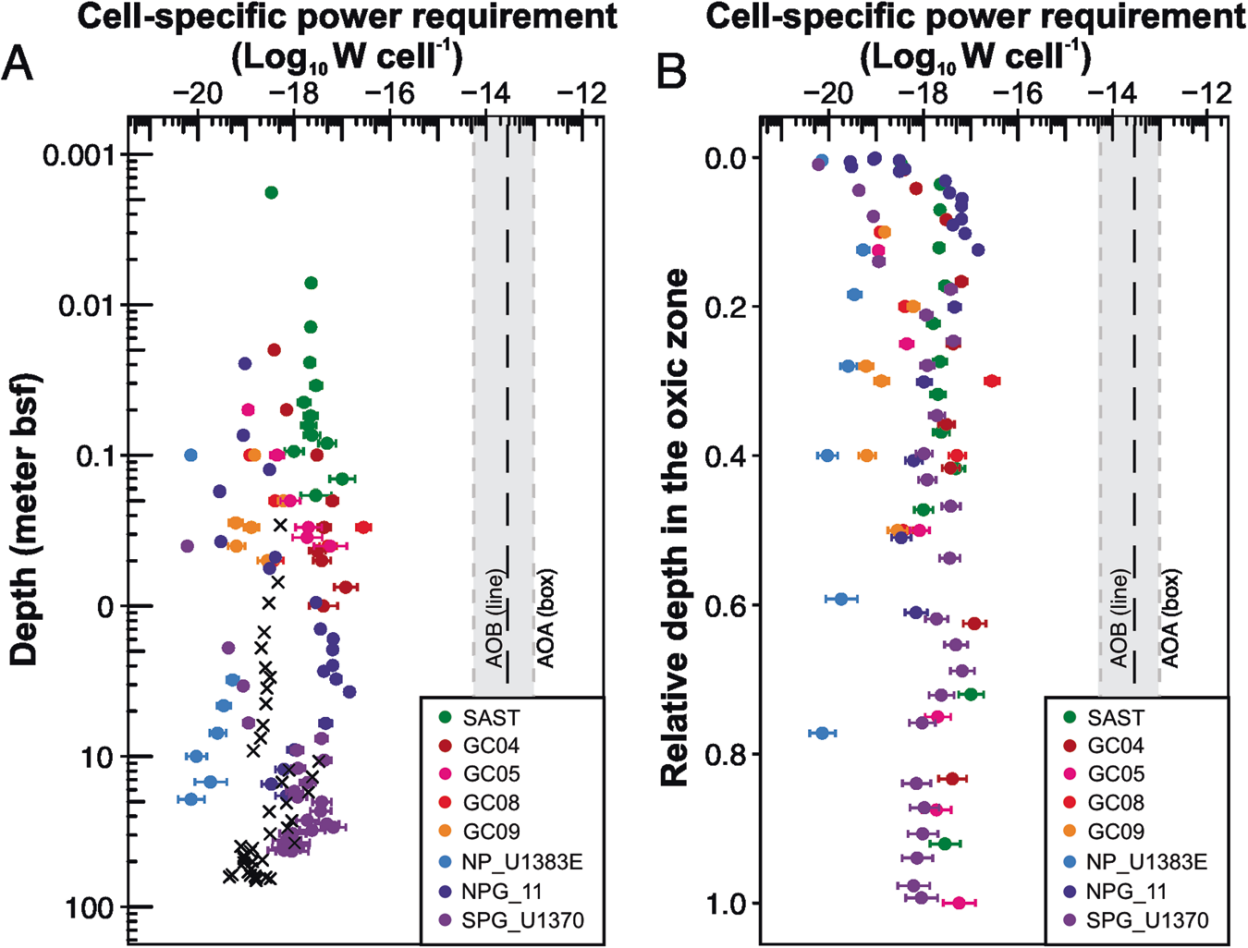
Cell-specific power requirement of AOA in marine oxic sediements. Cell-specific power requirement of AOA are plotted as a function of sediment depth (**A**) and of the relative depth in the oxic zone (**B**). Error bars represent the 95% of confidence level, derived from the AOA cell abundance estimation as presented in Fig. 4A. The cross symbol (×) in (**A**) represents the values calculated for bulk cells that were assumed to catalyze the aerobic degradation of POC in core SPG_U1370 [11]. In both panels, the gray box represents the cell-specific power requirement range calculated for AOA strained grown in the laboratory based on the cell-specific data reported in [29, 51, 52] (see “Materials and methods” for details). The vertical dashed line refers to the value of marine ammonia-oxidizing bacterium *Nitrosomonas marina* calculated by [10, 53] based on the chemostat experiment data reported in [54].

We calculated the cell-specific power requirement of AOA by assuming all the AOA cells have similar power requirements. This assumption is supported by the observation that AOA communities in the oxic sediments of NP_U1383E [23] and GC08 [31] are composed by a low diversity of Nitrosospumilaceae. We limited our calculation to the oxic zones because so far we lack a clear understanding of whether AOA detected below the oxic zones rely on ammonia oxidation for power supply, if they are indeed alive. The result shows that most of the calculated cell-specific power requirements of AOA are in the similar narrow range of 10^−19^–10^−17^ W cell^−1^ (Fig. 5A), regardless of sediment location. These values are lower than the lowest cell-specific power requirement of microbes measured in the laboratory (1.9 × 10^−17^ W cell^−1^ for the phototrophic bacterium *Chlorobium* BS‐1; [80]) but are well above the proposed minimum power requirement for a single cell (1 × 10^−21^ W cell^−1^ [10]). These cell-specific power requirements of AOA overlap with the range previously constrained for bulk microbial cells inhabiting oxic sediments (10^−19^−10^−16^ with a median of 2.23 × 10^−18^ W cell^−1^; [14]). This match is also particularly evident at SPG_U1370, where the cell-specific power requirements of AOA also generally match with those values estimated for the bulk cells (1.9 × 10^−19^ W cell^−1^; [11]) which were all assumed to catalyze the aerobic degradation of particulate organic carbon (Fig. 5A). The calculated cell-specific power requirements of AOA in marine oxic sediments are generally higher than those of sulfate reducers (with a median of 1.08 × 10^−19^ W cell^−1^) and methanogens (with a median of 1.50 × 10^−20^ W cell^−1^) in global anoxic sediments [14], consistent with previous compilations [16, 53]. This difference has been attributed to remarkably higher energetic costs of building block synthesis per cell in oxic compared to anoxic sediments [16, 81]. While most previous studies focused on heterotrophs [10, 14, 16], our study represents one of the first studies reporting the cell-specific power requirements of archaeal autotrophs in a wide range of marine sediments.

Cell-specific power requirements calculated for AOA in marine oxic sediments are two to six orders of magnitude lower than the values of AOA strains grown in the laboratory (calculated in the range of 5.6 × 10^−15^−8.8 × 10^−14^ W cell^−1^, based on the data reported in [29, 51, 52]) and the value estimated for ammonia-oxidizing bacterium *Nitrosomonas marina* grown under chemostat conditions (2.8 × 10^−14^ W cell^−1^, [53]) (Fig. 5A). This huge gap may suggest that the energy requirements of subseafloor microbial communities are too low to be mimicked by laboratory cultivation conditions that are often characterized as high-energy environments [2, 6, 16]. Our calculation also supports the notion that there are massive differences between microbial catabolism typically measured in the laboratory and those occurring in natural settings [10, 12, 16, 82–85].

### Similar cell-specific power requirements of AOA at different sediment depths

Our calculation of cell-specific power requirements provides a realistic constraint on BPR of AOA in the energy-limited subseafloor sediments. We observed that AOA inhabiting marine oxic sediments have similar power requirements spanning from the top centimeters to 42 mbsf (Fig. 5A) regardless of sediment properties, e.g., the seafloor organic matter flux, OPD, and sediment depth/age. In particular, the sediment core from the least active site, SPG_U1370, has the lowest nitrification rates and lowest AOA abundances, but the cell-specific power requirements of AOA at this site do not exhibit substantial differences from the other cores with higher activities and biomass (Fig. 5A). This range of cell-specific power requirements (10^−19^–10^−17^ W cell^−1^) could be very close to the BPR of AOA, which was thought to support the maintenance operation, like repair and replacement of damaged biomolecules, cross-membrane transport of ions, and nutrients and/or energy substrates [6, 16]. These observations suggest that AOA in the more energy-limited (deeper, older, and more oligotrophic) sediments are not necessarily facing more extreme conditions in terms of power requirement per cell.

When plotting the cell-specific power requirement against the relative depth in the oxic zone, we found a similar pattern across the eight cores: relative lower values in the uppermost part of the oxic zones, but increased to a relatively higher level (10^−18^–10^−17^) in the deeper part of the oxic zones (Fig. 5B). Similar increases were also reported previously for microorganisms catalyzing the knallgas reaction in SPG sediments [10]. Cell-specific extracellular enzyme activity of microbes in estuary sediments was also reported to increase with depth [86]. The increasing trend still holds in some cores (NPG_11, SPG_U1370, GC04, and GC05) after excluding the uppermost 10 cm, where the AOA population size may be not only constrained by power availability but also controlled by fauna grazing. Marine AOA are known to be infected by spindle-shaped viruses [87] of which sequences have also been detected in marine sediments [87]. Viral infection was also proposed as a key mechanism controlling the population turnover of AOA in deep-sea sediments [7]. The cell-specific power requirement increases could be partly attributed to the elevated viral infection pressure in marine sediments, as suggested by the increasing virus-to-cell ratio with depth in subseafloor sediments [8]. The activities of virus in marine sediments were also demonstrated by active viral production [88] and the expression of virus-related genes [9]. The viral activity may make cells in deeper and aged sediments undergo more viral infection than shallower sediments [6] and thus may result in higher cellular power requirements of prokaryotes in deep sediments.

The calculation of cell-specific power requirement requires both sediment geochemical data and microbial abundance data. While the latter type of data is still quite sparse from vast regions of the global seafloor, the former is available across a large scale of the global ocean (e.g., [21, 43, 44, 57–60, 89]). If the narrow range of the cell-specific power requirements of AOA estimated in this study can be extrapolated to other marine oxic sediments, calculating the total abundance of AOA relying on aerobic ammonium oxidation at certain sites can be carried out using geochemical data alone and estimating the standing stock of AOA in global marine sediments would be possible. Alternatively, if the above-described relationship between the relative abundance of AOA in the total community and the relative depth in the oxic zone is valid in other sites, the total AOA in marine oxic sediments could be more accurately estimated from the total cell numbers [1], in conjunction with the spatial and vertical distribution of O_2_ in the global seabed [5, 90].

## Conclusion

Our study provides quantitative insights into the energetics of AOA in marine oxic sediments, from the top millimeters to 42 meters below seafloor. By examining eight sediment cores with markedly different organic matter fluxes and OPDs, we showed that the reaction rate and power supply of nitrification exhibit a vertical decreasing trend in individual cores and also six-orders-of-magnitude variations between different cores. AOA abundance showed a log−log decreasing relationship with depth in marine oxic sediments, suggesting that this functional group is controlled by power ultimately derived from the degradation of organic matter.

The cell-specific power requirement of AOA is two to five orders of magnitude lower than the reported values for AOA and AOB obtained under laboratory conditions. The cell-specific power requirement of AOA varies in a narrow range throughout the examined sediment depths, and is similar across the five contrasting sites, providing a realistic constraint on the BPR of microbial life inhabiting subseafloor sediments. Combining with the abundant existing sediment geochemical data, our quantification of cell-specific power requirements of AOA may lay a foundation for a first-order estimate of the standing stock of AOA in the global marine oxic sediments.

## Supplementary information

Supplementary Information

## Data Availability

All data used in this study were previously published, which sources are listed in Table S1. The reaction-transport model is available at GitHub (https://github.com/ruizhao087/Reaction-Transport-Model-for-marine-sediments).
