# Supplementary material for: Ammonia-oxidizing archaea have similar power requirements in diverse marine oxic sediments
Source: ISME J. 2021 Jun 22;15(12):3657–67. doi: 10.1038/s41396-021-01041-6 (PMC8630020; doi:10.1038/s41396-021-01041-6)
Supplement: Supplementary file 1 — Supplementary Information [file 41396_2021_1041_MOESM1_ESM.docx]

**Supplementary Figures**

**
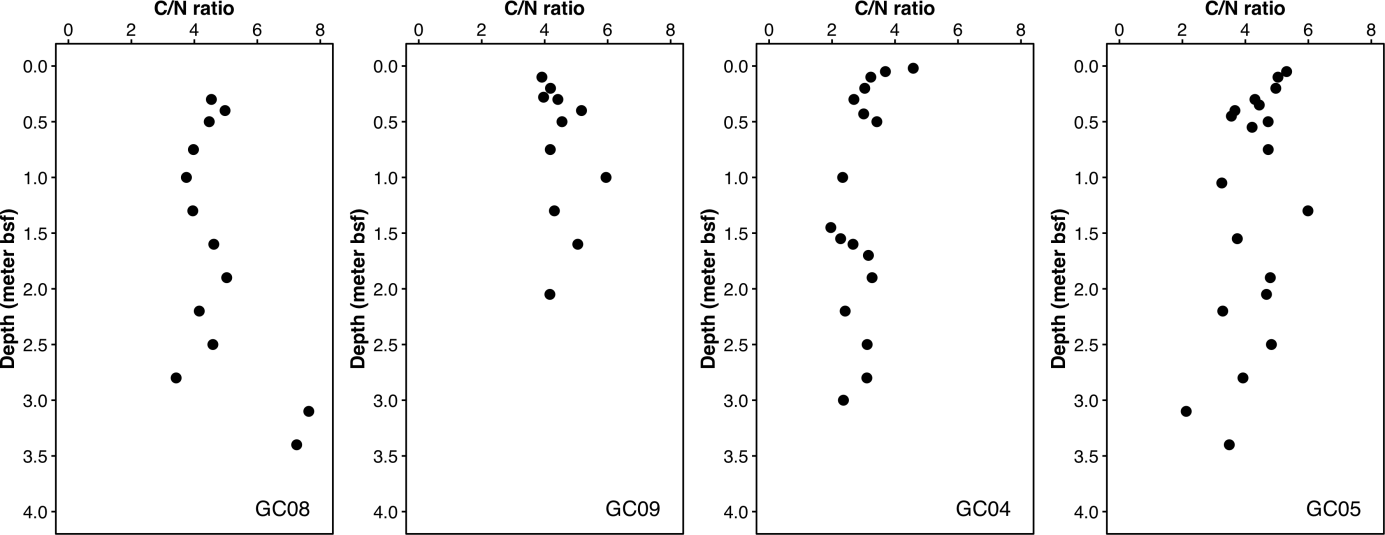
**

**Figure S1. Carbon to nitrogen ratio (C/N ratio) of organic matter measured in the AMOR sediment cores.**

**
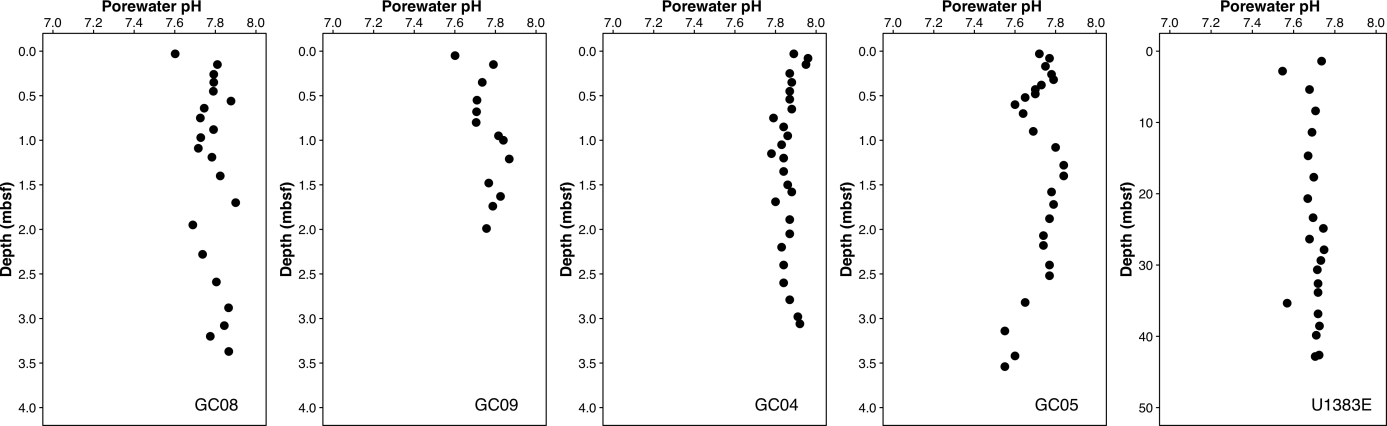
**

**Figure S2. Porewater pH measured in the AMOR sediment cores and NP_U1383E.**

**
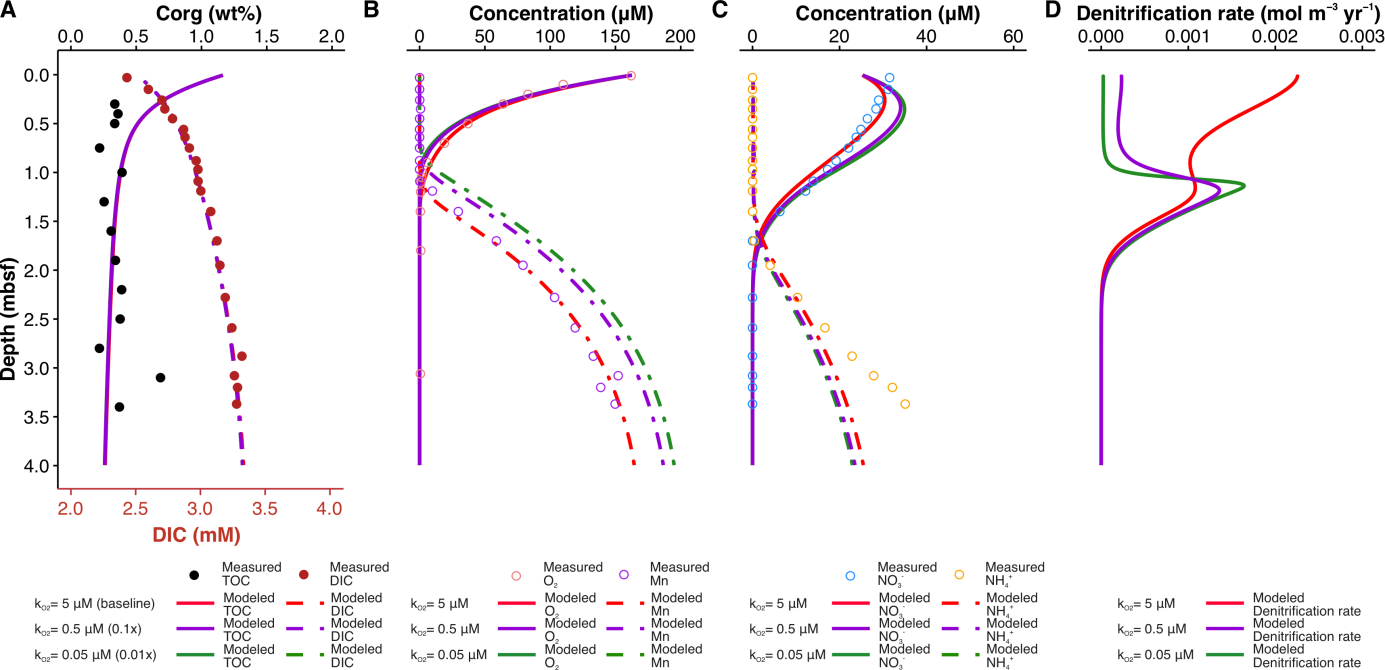
**

**Figure S3. Sensitivity analysis of the effect of the oxygen inhibition constant, k_O2_, on nitrate profile simulation.** Profiles showed here are the measured (dots) and modelled (lines) concentrations of TOC and DIC (A), O_2_ and dissolved Mn (B), NO_3_^-^ and NH_4_^+^ (C), and denitrification rate (D). All simulations were performed using the same model parameters, except for k_O2_, for which 5 µM (the baseline value), 0.5 µM (0.1x), and 0.05 µM (0.01x) were used.

**
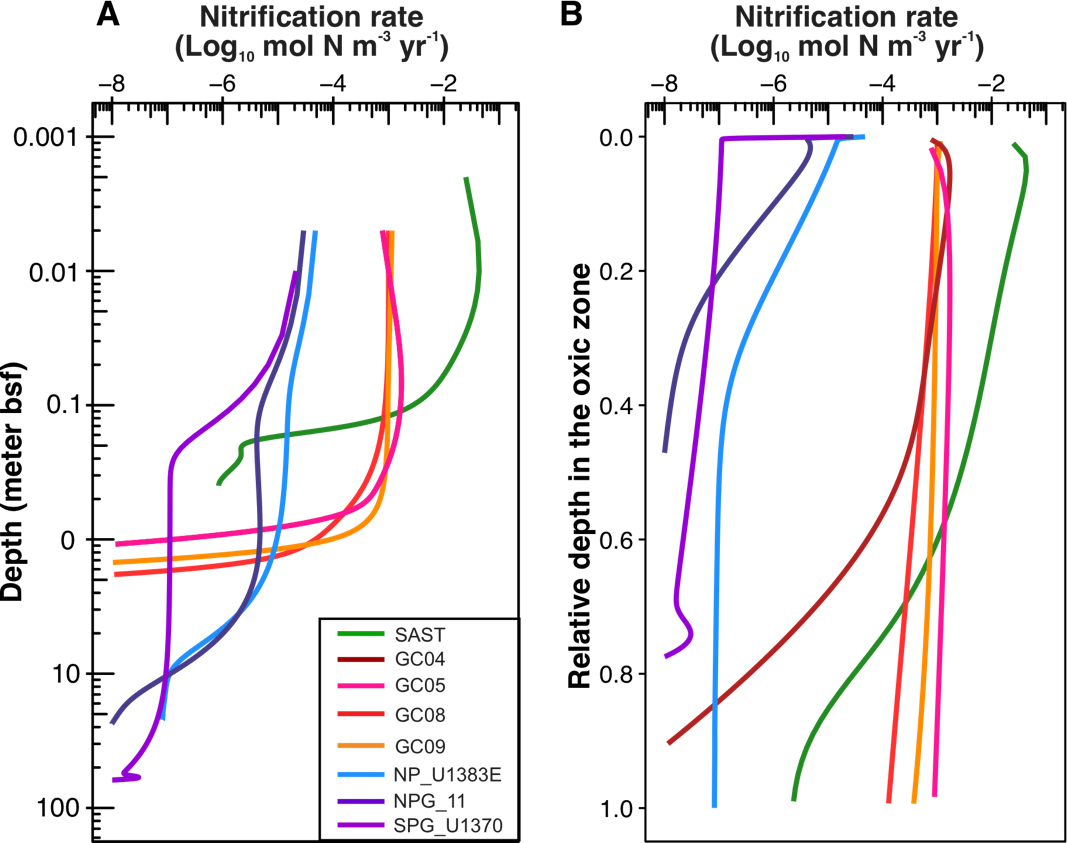
**

**Figure S4. Nitrification rates (log transformed) as a function of (A) log transformed depth and (B) the relative depth in the oxic zone in individual cores.**

**
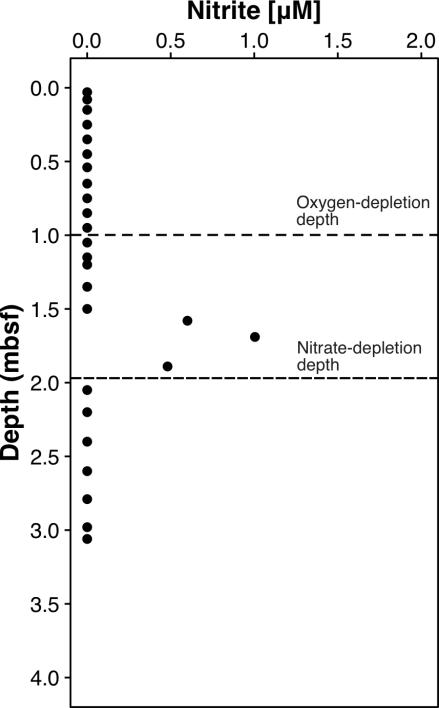
**

**Figure S5. Nitrite concentration in the sediment porewater of core GC04.** The two horizontal dashed lines represent the depletion depths of oxygen and nitrate. Note that the x-axis scale is different from that used for nitrate and ammonium in Fig. 2.


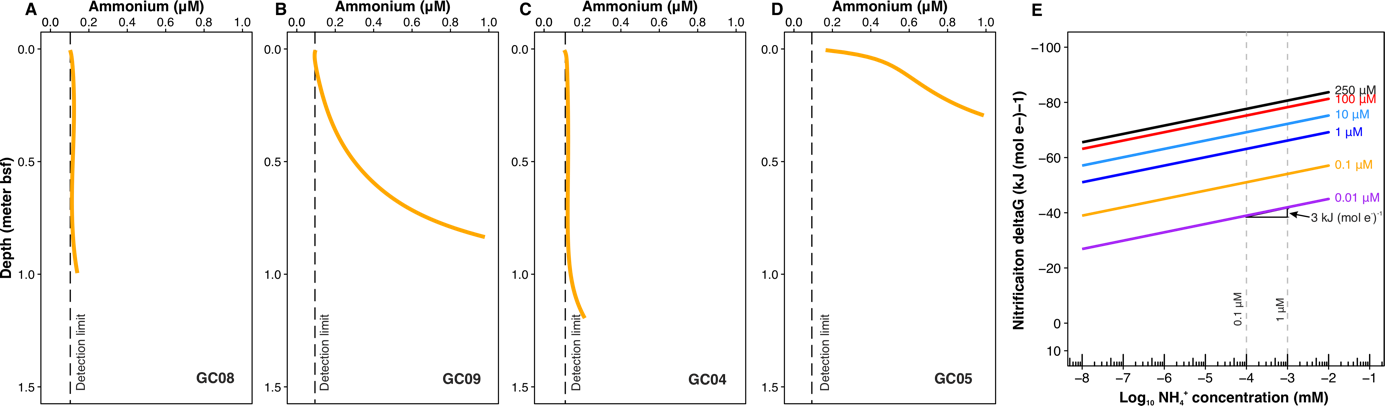


**Figure S6. Measured (dots) and modeled (lines) concentrations of ammonium in the oxic zone of the four AMOR cores (A-D) and their influences on the calculated Gibbs free energy of nitrification (E).** Note that ammonium concentrations were below the detection limit of 0.1 µM, but were assumed to be this value in the plots. The x-axis in panels **A**, **B**, **C**, and **D**, is in the range of 0-1 µM, lower than those used in Fig. 2. (**E**) Gibbs energy of nitrification for a wide range of oxygen (250, 100, 10, 1, 0.1, 0.01 µM) and ammonium concentrations. Also shown is the offset of Gibbs energy of nitrification at ammonium concentrations of 1 and 0.1 µM.


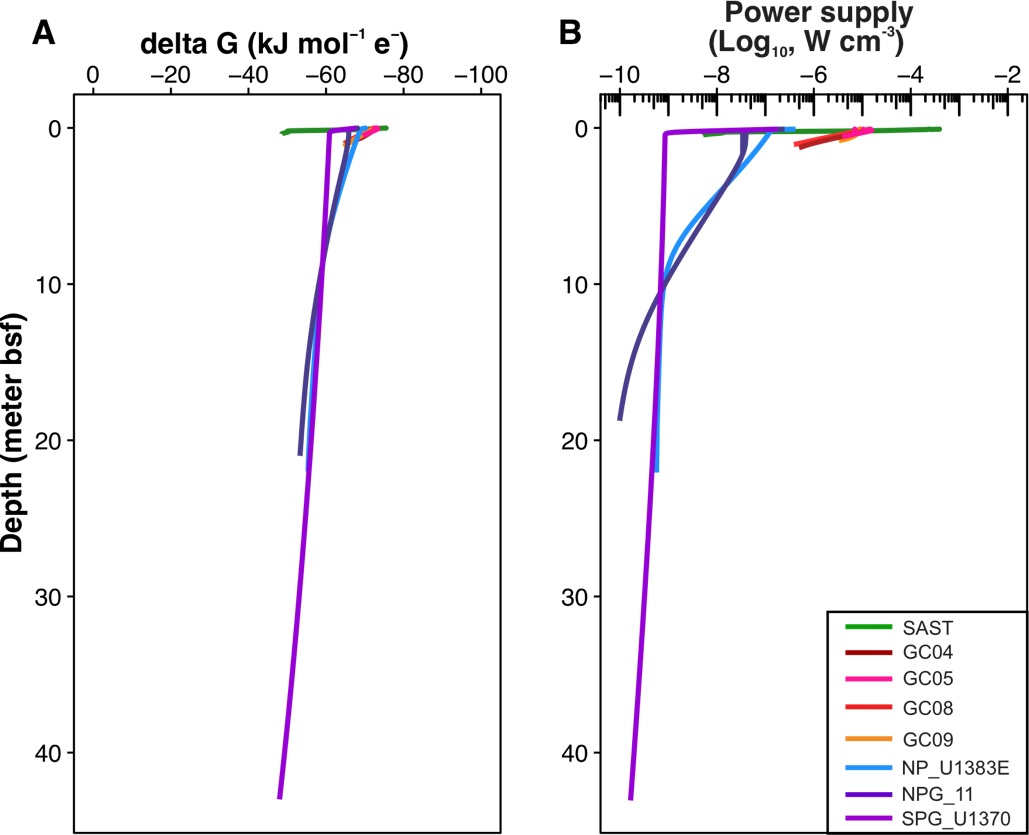


**Figure S7. Gibbs free energy (A) and power supply (B) of nitrification plotted against sediment depth.** Both are calculated using the modelled concentrations of relevant species from the reaction-transport model simulation, as presented in Fig. 2.


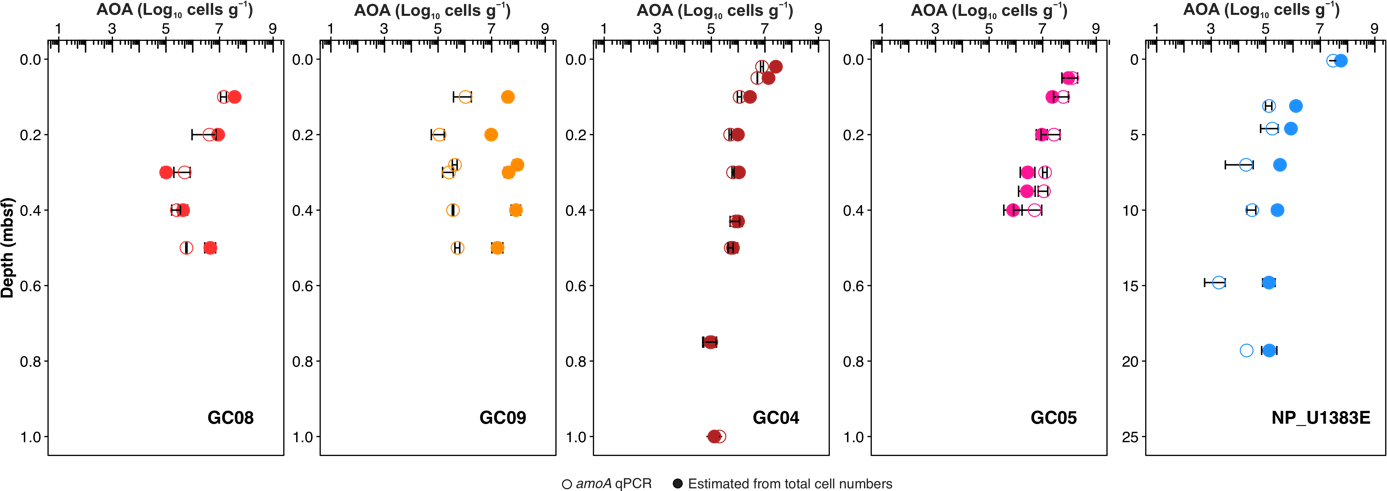


**Figure S8. Comparison of the abundances of AOA determined by qPCR of *amoA* gene and estimated from total cell numbers.** Open circles represent the abundances quantified by archaeal *amoA* gene-based qPCR, with the error bars represent the standard deviation of triplicate qPCR measurements. Filled circles denote the AOA abundances estimated from the total cell numbers, by assuming the fraction of AOA in total communities as presented in Fig. 4A. Error bars denote the 95% confidence interval of the estimates.

**Table S1. Data used to calibrate the model simulations and nitrifier abundance estimation**

| **Sediment core** | **Geochemical data (Porewater)** | **Geochemical data (Solid phase)** | **Microbiology data** | |
| --- | --- | --- | --- | --- |
| SAST | Oxygen^a^, Nitrate ^a^, Ammonium ^a^ | TOC ^a^ | Cell counts ^b^ | -- |
| GC04 | Oxygen^c^, Nitrate ^c^, Ammonium ^c^, Mn(II) ^c^, DIC ^c^ | TOC ^c^ | Cell abundance ^c^ | Functional group abundances ^c^ |
| GC05 | Oxygen^c^, Nitrate ^c^, Ammonium ^c^, Mn(II) ^c^, DIC ^c^ | TOC ^c^ | Cell abundance ^c^ | Functional group abundances ^c^ |
| GC08 | Oxygen ^c^, Nitrate ^c^, Ammonium ^c^, Mn(II) ^c^, DIC ^c^ | TOC ^c^ | Cell abundance ^c^ | Functional group abundances ^c^ |
| GC09 | Oxygen ^c^, Nitrate ^c^, Ammonium ^c^, Mn(II) ^c^, DIC ^c^ | TOC ^c^ | Cell abundance ^c^ | Functional group abundances ^c^ |
| NP_U1383E | Oxygen ^d^, Nitrate^e^, Ammonium | TOC^f^ | Cell abundance ^e^ | Functional group abundances ^e^ |
| NPG_11 | Oxygen ^g^, Nitrate ^h^ | -- | Cell counts ^g^ | -- |
| SPG_U1370 | Oxygen ^i^, Nitrate ^i^, DIC ^i^ | TOC ^i^ | Cell counts ^i^ | -- |

a, ref. [33];

b, ref. [32];

c, ref. [13];

d, ref. [34];

e, ref. [23];

f, ref. [35];

g, ref. [4];

h, ref. [36];

i, ref. [5];

--, not available.

**Table S2. Species and boundary conditions (BC) at the sediment-water interface (SWI) used in the reaction-transport model**

| Name | Symbol | BC SWI Type (Unit) | BS SWI Value | | | | | | | |
| --- | --- | --- | --- | --- | --- | --- | --- | --- | --- | --- |
|  |  |  | SAST | GC04 | GC05 | GC08 | GC09 | NP_  U1383E | NPG_11 | SPG_U1370 |
| Total organic carbon | CH_2_O | Flux (mol m^-2^ yr^-1^) | 2.0E-1 | 1.09E-2 | 2.01E-2 | 9.30E-3 | 1.42E-2 | 2.3E-3 | 6E-4 | 1.24E-4 |
| Oxygen | O_2_ | Concentration (µM) | 150 | 205 | 160 | 165 | 225 | 250 | 150 | 130 |
| Ammonium | NH_4_^+^ | Concentration (µM) | 0.1 | 0.1 | 0.1 | 0.1 | 0.1 | 0.01 | 0.01 | 0.01 |
| Nitrate | NO_3_^-^ | Concentration (µM) | 35 | 21 | 30 | 25 | 21 | 21 | 36 | 40 |

**Table S3. Parameter values used in the reaction-transport model**

| Name | Symbol | Unit | SAST | AMOR GC04 | AMOR GC05 | AMOR GC08 | AMOR GC09 | NP_U1383E | NPG_11 | SPG_U1370 |
| --- | --- | --- | --- | --- | --- | --- | --- | --- | --- | --- |
| Sediment domain | L | cm | 40 | 500 | 600 | 500 | 350 | 2200 | 3000 | 7000 |
| Solid burial velocity at compaction | ω | cm ky^-1^ | 1.2 | 2 | 2.5 | 2 | 5 | 0.94 | 0.1 | 0.15 |
| TOC degradation constant C_1_ | kfox | 1 yr^-1^ | 0.9 | 6.5E-5 | 9.0E-5 | 3E-5 | 6.5E-5 | 5.6E-6 | 2.4E-7 | 7.0E-8 |
| TOC degradation constant C_2_ | kfox2 | 1 yr^-1^ | 0.003 | 2.0E-5 | 8.0E-6 | 1E-6 | 2.0E-5 | 4.2E-8 | 3.0E-8 | 3.5E-8 |
| Nitrification rate constant | *k*_4_ | µM^-1^ yr^-1^ | 300 | 300 | 150 | 150 | 150 | 150 | 150 | 150 |
| Mn oxidation rate constant | *k*_5_ | mM^-1^ yr^-1^ | -- | 110 | 110 | 110 | 110 | -- | -- | -- |
| Bioturbation coefficient | *D*_b,0_ | cm yr^-1^ | 1 | 0 | 0 | 0 | 0 | 0 | 0 | 0 |
| Biomixing half depth | z_mix_ | cm | 3 | 3 | 3 | 3 | 3 | 3 | 3 | 3 |
| Biomixing attenuation | Z_att_ | cm | 3 | 3 | 3 | 3 | 3 | 3 | 3 | 3 |
| Bioirrigation coeffcient | *α*_0_ | yr^-1^ | 1 | 0 | 0 | 0 | 0 | 0 | 0 | 0 |
| *R*_1_ O_2_ inhibition concentration | *h*_1_ | µM | 10 | 15 | 10 | 5 | 10 | 2 | 4 | 0.2 |
| *R*_2_ NO_3_^-^ inhibition concentration | *h*_2_ | µM | 30 | 4 | 5 | 10 | 10 | 1 | 1 | 1 |
| *R*_3_ MnO_2_ inhibition concentration | *h*_3_ | µmol g^-1^ | -- | 10 | 10 | 10 | 10 | -- | -- | -- |

--: not used.

**Table S4. Root mean square error (RMSE) of porewater solutes^a^**

| **Core ID** | **O_2_**  **[µM]** | **Mn(II)**  **[µM]** | **NO_3_^-^**  **[µM]** | **NH_4_^+^**  **[µM]** | **DIC**  **[mM]** |
| --- | --- | --- | --- | --- | --- |
| SAST | 8.8 | -- | 2.8 | -- | -- |
| GC04 | 7.9 | 14.3 | 2.3 | 0.5 | 0.07 |
| GC05 | 8.6 | 8.0 | 1.1 | 0.5 | 0.07 |
| GC08 | 8.1 | 3.7 | 1.8 | 4.6 | 0.03 |
| GC09 | 4.7 | 7.9 | 3.1 | 1.2 | 0.07 |
| NP_U1383E | 19.6 | -- | 2.4 | -- | -- |
| NPG_11 | 12.2 | -- | 1.6 | -- | -- |
| SPG_U1370 | 9.4 | -- | 1.0 | -- | -- |

^a^ Error is calculated not for any single data point, but for the whole simulated concentration profile.

--: not used.
